# Conjugated linoleic acid loaded nanostructured lipid carrier as a potential antioxidant nanocarrier for food applications

**DOI:** 10.1002/fsn3.1712

**Published:** 2020-06-15

**Authors:** Fatemeh Sadat Hashemi, Farin Farzadnia, Abdoreza Aghajani, Farnaz Ahmadzadeh NobariAzar, Akram Pezeshki

**Affiliations:** ^1^ Department of Food Science and Technology Elmi‐karbordi University of Samin Nan Sahar Tehran Iran; ^2^ Department of Food Science and Technology Faculty of Agriculture Mamaghan Branch Islamic Azad University of Mamaghan Mamaghan Iran; ^3^ Department of Food Science and Technology Faculty of Industrial and Mechanical Engineering Qazvin Branch Islamic Azad University Qazvin Iran; ^4^ Department of Food Science and Technology Faculty of Agriculture University of Tabriz Tabriz Iran

**Keywords:** conjugated linoleic acid, gas chromatography, low‐fat milk, nano lipid carrier, particle size

## Abstract

The encapsulation of fatty acids in nanocarrier systems is a very effective technique in improving their biological efficiency and controlled delivery. Nanostructured lipid carrier (NLC) is a major type of lipid‐based nanoparticle. This study is focused on producing nanolipid carrier containing conjugated linoleic acid and fortifying low‐fat milk using this nanoparticle. Nanostructured lipid carriers were produced by hot high‐shear homogenization containing 1.5% Poloxamer 407, cocoa butter as solid lipid, and conjugated linoleic acid as liquid oil in ratio of 10:1. Results showed that the nanoparticles sized 81 nm with monomodular dispersity and the system was stable at 4 and 22°C for 40 days. Zeta potential and encapsulation efficiency (%EE) were −15.8 mV and 98.2%, respectively. Scanning electron microscopy (SEM) showed that the particles are in spiral form and small size and no significant aggregation was observed because of few changes in the system turbidity after storage time. The result of oxidative stability showed that using Nanostructured lipid carriers system resulted in lower malone dialdehyde production. Conjugated linoleic acid was protected at level of 3.9% of milk fatty acids in Nanostructured lipid carrier formulation during storage time. Based on these findings, Nanostructured lipid carriers system is an appropriate and stable nanocarrier system for delivery of nutraceuticals in foods and can be used in protecting them against oxidation, heating, and other processes in order to fortify foods and beverages.

## Graphical abstract

Nanostructured lipid carrier production was done by hot high‐shear homogenization containing 1.5% poloxamer 407, cocoa butter as solid lipid, and conjugated linoleic acid as liquid oil in ratio of 10:1. Results showed that the nanoparticle size was 81 nm with monomodular dispersity and the system was stable at 25°C for 60 days.

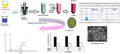

## 1 INTRODUCTION

Milk and dairy products are highly nutritious food for individuals throughout all stages of life. The composition of milk directly influences the quality, yield, functionality, and nutritional value of milk and dairy products (O'Callaghan et al., 2019). Milk is a rich source of nutrients and considered by many as a valuable component of a perfect diet. Milk contains many bioactive components that boost the physiological processes in the body (Khush, 2002). Milk fat plays an important role in quality of milk and dairy products, nutritional value, and diet. The premium nutritional quality of dairy products is highly correlated with milk fat quality and concerns high concentration of fat‐soluble vitamins and n‐3 fatty acids, as well as high content of conjugated linoleic acid (CLA). Moreover, milk fat influences processing of raw material and is a carrier of taste and aroma (Cieslak, Kowalczyk, Czauderna, Potkanski, & Szumacher‐Strabel, 2010; Jozwik, Strzalkowska, Bagnicka, Polawska, & Horbanczuk, 2010; Strzalkowska et al., 2010). The linoleic acid (LA) present in milk is known as a potential anticarcinogen, which can be controlled through diet management (Morsy et al., 2018). Diet is the major source of milk CLA and many trials have been conducted with the aim of enhancing milk CLA content (Tripathi, 2014). CLA refers to a group of positional and geometric isomers of linoleic acid that are characterized by the presence of conjugated dienes. CLA is a natural, but minor, component of fats from ruminant animals that enters the human diet primarily through meat and dairy products (Whigham, Watras, & Schoeller, 2007). CLA is considered as an essential fatty acid which represents a group of linoleic acid isomers (18:2) each with intensive biological functions such as anticancer, anti‐obesity, and anti‐hypertension (MacDonald, 2000). Drug delivery systems have opened new avenues to improve the therapeutic effects of already‐efficient molecules. Some drugs are poorly soluble in water and cannot be administered unless they are encapsulated as drug carriers. In other occasions, drugs cannot permeate cell membranes and as a consequence the concentration at the target site is insufficient. To overcome this, high doses of drugs are required, causing high toxicity and many undesired side effects (Limeres, Moretton, Bernabeu, Chiappetta, & Cuestas, 2019). Consequently, a targeted drug delivery system could selectively carry sufficient drug concentrations into the targeted tissue (or cell) improving its bioavailability and reducing the associated side effects due to high doses (Bayon‐Cordero, Alkorta, & Arana, 2019).

Poloxamer (also known as “Pluronic”) is a triblock amphiphilic copolymer of ethylene oxide (EO) and propylene oxide (PO) (Shubhra, Toth, Gyenis, & Feczko, 2014). Different poloxamer excipients have been extensively used in pharmaceutical industries. They are used as emulsifier (Feczko, Toth, & Gyenis, 2008), solubilizer for hydrophobic drugs (Shah, Amin, Parikh, & Parikh, 2007), and suspension stabilizer (Wulff‐Perez, Torcello‐Gomez, Martin‐Rodriguez, Galvez‐Ruiz, & de Vicente, 2011). Poloxamer 407 in combination with a liposome showed an increase in stability of liposome formulation by increasing half‐life, preventing aggregation, and fusion of phosphatidylcholine multilamellar vesicles (Nogueiras‐Nieto, Sobarzo‐Sanchez, Gomez‐Amoza, & Otero‐Espinar, 2012). The low stability of poloxamer hydrogel in an aqueous solution leads to the development of combined poloxamer 407 with acrylate and thiol groups as 17.5 wt% at body temperature. It was observed with an immediate crosslinking formed between acrylate and thiol that modified poloxamer 407 property, giving rise to a remarkable increase in stability of drugs about four times and for its potential application in controlled drug release (Pezeshki, Ghanbarzadeh, Mohammadi, Fathollahi, & Hamishehkar, 2014). Along with the development of nanotechnology, its applications in the medical and health sciences have increased dramatically. Recently, researchers widely use metallic nanoparticles (Shaabani, Amini, Kharrazi, Tajerian, & Curcumin, 2017; Shirkhanloo, Osanloo, Ghazaghi, & Hassani, 2017), polymeric nanoparticles and nanofilms (Abyadeh et al., 2017; Firoozi, Amani, Derakhshan, & Ghanbari, 2016; Gholami, Ahmadi, & Ahmadi, 2020), and lipid‐based nanoformulations (e.g., nanoemulsions, nanocubosomes, solid lipid nanoparticles (SLNs), nanostructured lipid carriers (NLCs) and liposomes) (Akhtari et al., 2016; Osanloo, Amani, et al., 2017; Osanloo, Sereshti, Sedaghat, & Amani, 2017; Radbeh, Asefi, Hamishehkar, Roufegarinejad, & Pezeshki, 2020) as drug carriers. Carrier is a special molecule or system used for the effective transportation of a loaded drug to preselected sites to serve targeted drug delivery. Carriers are engineered vectors, which retain drugs either on the cell surface or in a subcellular compartment via physical or chemical interaction, encapsulation, and spacer moiety (Alavizadeh, Akhtari, Badiee, Golmohammadzadeh, & Jaafari, 2016).

One of the nanocarriers is nanostructured lipid carrier (NLC) with a unique structure and specific benefits such as high encapsulation efficiency (EE), stability against gravitational separation (due to highly particle density) and low release rate (Beloqui, Solinis, Rodriguez‐Gascon, Almeida, & Preat, 2016; Brar & Verma, 2011). In order to compensate the lack of unsaturated fatty acids in foods, encapsulation by NLCs would be recommended. Lipid nanoparticles have many advantages in comparison to other particulate systems including ease of large scale production (Luo, Chen, Ren, Zhao, & Qin, 2006), biocompatible and biodegradable nature of the materials (Silva et al., 2011), low toxicity potential (Ghasemiyeh & Mohammadi‐Samani, 2018), possibility of controlled and modified drug release, drug solubility enhancement and the possibility of both hydrophilic and lipophilic drug incorporation (Zur Muhlen, Schwarz, & Mehnert, 1998). NLCs have remarkably wide range of properties which make them useful for parenteral, dermal, pulmonary, and topical delivery of drugs. These products have been developed in order to reduce toxic side effects of the incorporated highly potent drugs and increase the efficacy of the treatment (Naseri, Valizadeh, & Zakeri‐Milani, 2015). NLCs are second generation of lipid‐based nanocarriers formed from mixture of solid and liquid lipids and have unstructured matrix due to the different moieties of the constituents of NLCs (Beloqui et al., 2016). Use of nutraceuticals compounds in food products is often restricted by the low stability of them against environment condition. So for human health, food fortification is very crucial (Karimi, Ghanbarzadeh, Hamishehkar, Mehramuz, & SamadiKafil, 2018; Pezeshky, Ghanbarzadeh, Hamishehkar, Moghadam, & Babazadeh, 2016) and production of functional ingredients has get an abundant attention in food industry. In recent years, using nanocarriers is a positive approach for food fortification, which can be effective in delivery and stability of hydrophobic compounds (Fathi & Varshosaz, 2013; Gonnet, Lethuaut, & Boury, 2010), also during the digestion nanocarriers offer higher bioavailability and inhibit the developement of off‐flavor and off‐color of the food (Mehmood, 2015; Sagalowicz & Leser, 2010; Shukla et al., 2014),

The purpose of this study was to encapsulate a labile lipophilic compound, CLA, into NLC using hot high‐shear homogenization (Hot‐HSH) The particle size of NLC systems and physical stability of the NLC systems was evaluated over a storage period of 60 days and the encapsulation efficiency (EE %) and loading capacity (LC, %) were obtained. In final survey, the functional properties of CLA‐NLC in transferring, releasing, and protecting CLA in pasteurized low‐fat milk were studied.

## 2 MATERIALS AND METHODS

### 2.1 Materials

CLA with a purity of 80%, a mixture of isomers 9‐cis, 11‐trans and 10‐trans, 12‐cis (Zahravi pharmaceutical Co.), law‐fat pasteurized milk (>0.6% fat) (Pegah Co.). Cocoa butter (CB) (Shirin Asal Co.) and ploxamer 407, were purchased from Sigma Aldrich and all chemical in order to test were analytical grade and obtained from Merck Chemical Co.

### 2.2 Preparation of NLC

Making of NLC was done by hot high‐shear homogenization (Hot‐HSH) (Heidolph Instruments GmbH and Co.) at 22000 *g* (Keivani Nahr, Ghanbarzadeh, Hamishehkar, & Samadi Kafil, 2018). At first, the lipid phase included CB and CLA in specific weight (in ratio of 10:1) in aqueous bath were heated to 80°C in a hot water bath. Then, the aqueous solution containing 1.5% Ploxamer 407 was heated to 80°C and added drop by drop into the lipid phase while being homogenized. For recrystallization of the lipid phase and form NLC, (CLA + CB)/w nanoemulsion was kept at 22°C.

### 2.3 Particle size and zeta potential measurements

Wing SALD 2101 particle size analyzer (Shimadzo Corp.) was used for measurement the average volume diameter (DeBroukere mean particle size) and particle size distribution (span) of the particles at 22°C. Zeta potential of CLA loaded NLC was measured using zetasizer (Malvern Instruments) on first day after production.

### 2.4 Loading parameters

To obtain the Encapsulation Efficiency (EE %) and loading capacity (LC, %), amount of free CLA was separated from encapsulated CLA using an ultrafiltration method (Amicon Ultracentrifugal filter—nominal cut off of 30,000 Da) with centrifugation. NLC was regularly mixed with 50% w/w ethanol (in the ratio of 1:6), followed by centrifugation for 10 min at 2000 *g* (Universal 320, Camlab CO.). The filtrate was diluted with 50% w/w ethanol and measured using an Ultrospec 2000 spectrophotometer (Scinteck Co.) at 22°C at *λ* = 233 nm. EE and LC, % were calculated according to the following Equations (1) and (2): (Babazadeh, Ghanbarzadeh, & Hamishehkar, 2017):

(1) $$\%\text{EE}=\frac{\text{Total}\;\text{CLA}-\text{free}\;\text{CLA}}{\text{Total}\;\text{CLA}}\times 100$$

(2) $$\text{\textbackslash{}\% LC}=\frac{\text{incorporated amount of CLA}}{\text{Total of used lipid}}\times 100$$

### 2.5 Scanning electron microscopy (SEM)

The surface morphologies of obtained NLC at 1st after production and after 40th days of storage were investigated using SEM (KYKY‐EM3200 with an accelerating voltage of 26 kV). Before scanning, samples were diluted 20 times with deionized water (Klang, Matsko, Valenta, & Hofer, 2012).

### 2.6 Physical stability

In order to checking physical stability of NLC system during storage, particle size changes and the physical appearance of the NLC formulation during storage at 4 and 22°C for 40 days (on days 1, 7, 14, 30 and 40th day) were studied (Mohammadi, Pezeshki, Abbasi, Ghanbarzadeh, & Hamishehkar, 2017).

### 2.7 Turbidity assessment

The turbidimeter apparatus (Hach 2100p) with a series of turbidity standards in the range of 0–1,000 NTU (Nephelometric Turbidity Units) was used for turbidity measurement of diluted NLC formulations (diluted 10 times with water) in first, 30th and 60th days after production. The original sample turbidity obtained using Equation (3):

(3) $$\text{NTU}=\frac{\left(\text{NTU sample}*\left(\text{volume of dilution water}+\text{sample volume taken for dilution}\;\left(\text{ml}\right)\right)\right)}{\text{sample volume taken for dilution}\;\left(\text{ml}\right)}$$

### 2.8 CLA oxidative stabilization

Oxidative stability of NLC containing CLA was done using tiobarbituric acid (TBA) test during 30, 45, and 60th days of storage. 1 ml of NLC sample reagent with TBA solution (34 g of trichloroacetic acids 1.76 ml of 12 M hydrochloric acid and 8.28 ml water).

### 2.9 Fourier‐transform infrared spectroscopy (FTIR)

The infrared spectra were scanned using the FTIR spectrophotometer (IRAffinity‐1S, Shimadzo) with the sample:KBr ratio of 1:10 at 4 cm^−1^ resolution within the frequency ranges between 4,000 and 400 cm^−1^ (one scan per single outcome) (Keivani Nahr et al., 2018).

### 2.10 DPPH scavenging assay

The free radical scavenging capacity of CLA‐NLC was calculated using the methodology of Soleimanian, Golia, Varshosaz, & Sahafi (2018). At first 2 ml of DPPH solution (0.1 mM in ethanol) was added to 0.3 ml of NLC ethanolic solution (500 ppm) and allowed to react at room temperature. After 30 min, the absorbance values were measured at 517 nm against the blank sample (0.1 mM ethanolic DPPH solution). The radical scavenging activity (inhibition percentage) was stated as percentage of DPPH radical elimination calculated according to the following equation (4)

(4)

$$\text{Inhibition percentage}\;\left(\%\right)=\left(A\;\text{control}-A\;\text{sample}/A\;\text{control}\right)\times 100$$

where A control is the absorbance value of blank and A sample is the absorbance value of the sample at 517 nm.

### 2.11 CLA stability in pasteurized milk

#### 2.11.1 Preparation of enriched milk samples

Due to the 2.5 µg/ml CLA enrich to low‐fat milk, 177 µl of NLC formulation, 250 µg of pure CLA without nanocarrier (control sample) was added to 100 ml raw milk samples in two tubes, separately, and then were pasteurized in 65°C for 30 min. In order to extract fatty acid profile from milk, sampling was done from pasteurized and raw milk.

#### 2.11.2 Fatty acid profiles measurement in milk

Identifying and determining the extracted fatty acids were fulfilled using Autosystem XL GC (Perkin Elmer) with UV ionization detector (GC‐FID) at the following condition was used for separation, detection, and analysis of fatty acid available: capillary column, silica, 100 m length, 0.25 mm inner diameter; N2 (purity = 99.8%) used as carrier gas with a flow rate of 2 ml/min. Splitless mode injection was 50 ml/min splitting ratio in 0.75 min. The initial column temperature was 100°C and then the temperature was increased to 240°C at the 2°C/min and kept at 240°C for 15 min. The injection site temperature and detector were set on 270 and 300°C, respectively.

### 2.12 Statistical analysis

One‐way ANOVA (based on a complete randomized design) and Duncan's mean comparison tests (*p* < .05) were used with IBM SPSS Statistics for Windows, version 19.0 (IBM Corp., Armonk, NY, USA) for statistical analysis of results. Data were analyzed by SPSS software version 24. The comparison of the mean of data was done with Duncan's test and at the 5% (*p* < .05) probability level. All graphs are plotted with Excel 2013 software. The set of data was normalized using SPSS version 19 software (IBM Corp., Armonk, NY, USA) and all data was normal.

## 3 RESULTS AND DISCUSSION

### 3.1 Size particle, zeta potential and loading parameters (EE and LC%) measurements

**FIGURE 1:**
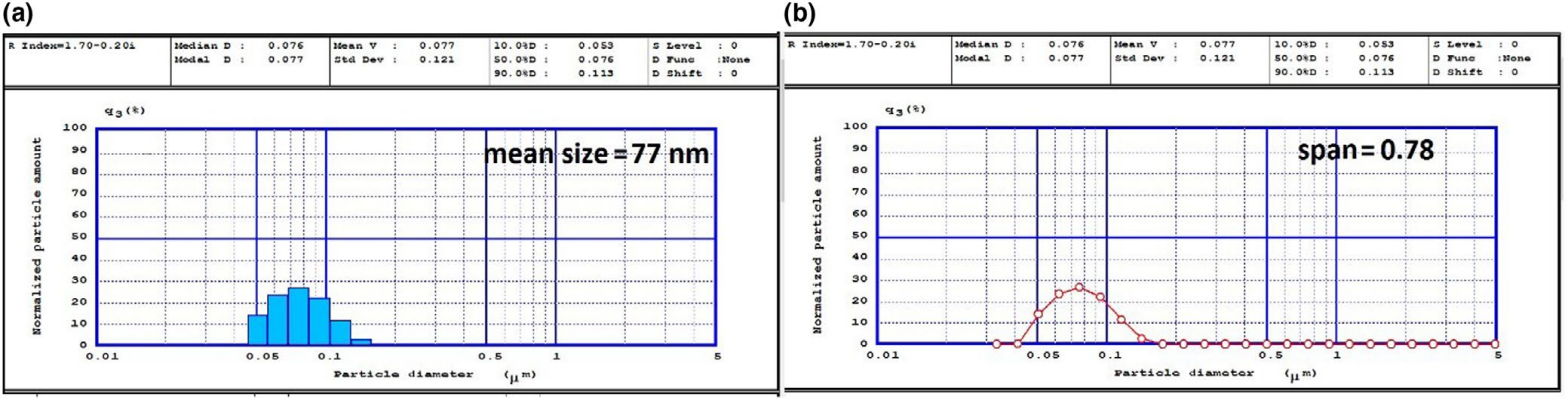
(a) Particle size and (b) particle size distribution 1 day after production of NLC formulation in ratio 10:1 solid liquid (cocoa butter) into liquid oil (CLA) using 1.5% (w/v) aqueous surfactant (Poloxamer407.)

The average particle size (*D*_4,3_), particle size distribution (span), zeta potential, EE, and LC % of the CLA bearing NLC formulation, on first day after production were shown in Table 1. The results showed the appropriate distributing and uniformity of particle size in nanoscale in NLC system (Figure 1). So in this research, zeta potential value of NLC formulation might be due to the composition of CLA and the presence of electrical barrier on the particles surface is responsible for the physical stability of nanodispersions by repulsion phenomenon Table 1. The EE and LC% of CLA in NLC system were 98% and 8.9%, respectively. The high %EE can be due to its lipophilic properties. The solid lipid to liquid oil ratio has an important effect on the EE and LC% of the NLC. There is an equilibrium between the content of solid lipid and liquid oil. Once the balance is broken, it is not advantageous for the maximum formation of lipid carriers with imperfect crystal, which had a large amount of space to load more bioactive substances (Pezeshki et al., 2019).

**TABLE 1:** The average particle size (*D*_4,3_), particle size distribution (span) and zeta potential of the CLA bearing NLC

Formulation	Size diameter (nm)	Span	Zeta potential (mV)	EE (%)	LC (%)
NLC	77	0.78	−12.3	98.2	8.9

Instability can result from interaction between poorly charged or uncharged nanoparticles, leading to the formation of aggregates. Zeta potential is a fundamental particle characteristic that can also be rapidly measured using light‐scattering techniques (Brar & Verma, 2011). Zeta potential measurements provide precise analysis of the electronic state of the nanoparticle surface, and the data obtained can be used to predict the stability of formulations containing these nanoparticles (Kaszuba, Corbett, Watson, & Jones, 2010). The small size of developed colloidal systems confirms good compatibility among compensates (Karimi et al., 2015). Choosing proper lipid matrix and the type of surfactant is an important key factor in preparing a stable and proper nanocarrier. It might be noted that use many different types of solid lipid for preparing NLC such as natural lipid (CB), semi‐synthetic, and synthetic (Precirol ATO5) with various structures. Selecting a proper ratio from lipid and oil also plays a crucial role to create a crystal structure and uniform particle size distribution in a stable system (Soleimanian et al., 2018). At low concentrations of liquid oil, oil molecules would be spread solid lipid matrix and NLC will have an incomplete structure. In formulation which containing high concentration of liquid oil, the matrix of liquid oil in solid lipid structure would decrease during cooling phase separation happens, the size and distribution of particles will increase (Tamjidi, Shahedi, Varshosaz, & Nasirpour, 2013).

Due to polymeric and bulky structure of Poloxamer 407 in NLC formulation, the stability of the nanoparticles within the storage time could be due to the steric repulsion of surfactant molecules rather than electrostatic repulsion. Trotta, Debernardi, and Caputo (2003) stated that the stability of the nanolipid carrier against aggregation is influenced by the ionic strength of the continuous phase and the charge density on the surface of water and fat. EE % of CLA in NLC system was more than 90%. The high %EE can be due to its lipophilic properties, which leads to its higher ability into lipid matrix compared to aqueous phase (Ni, Zheng, Hara, Pan, & Luo, 2015). Similar results were gained by other researchers (Babazadeh et al., 2017; Keivani Nahr et al., 2018; Pezeshki et al., 2019), with high EE % more than 90% in all formulations.

### 3.2 SEM image

**FIGURE 2:**
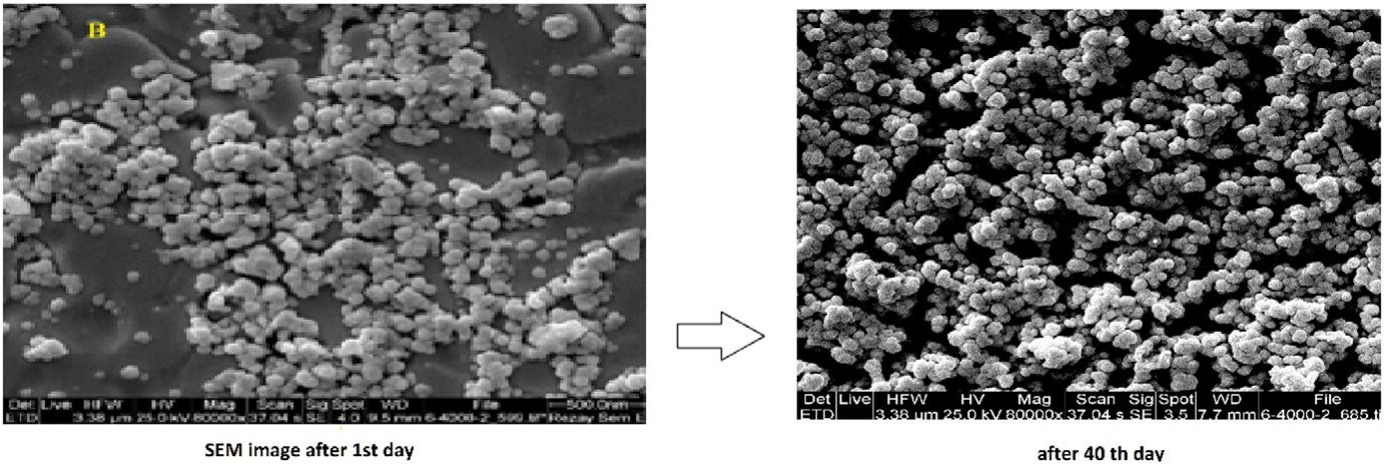
SEM morphology of CLA loaded NLC at 1st and 40th days of storage time

SEM was used to get the visual information regarding the morphology and size of NLC. SEM images at 1st and 40th days of storage time showed small size of spherical and homogeneous particles with smooth surface in nanocarrier systems, without occurrence of phase separation, aggregation. Also, the results of the size measurements of nanoparticles obtained from measurement devices and their stability during storage time were confirmed (Figure 2).

Scanning electron microscopy (SEM) uses electrons for imaging, in much the same way that a light microscope uses visible light, with the main improvements including greater depth of field and higher magnification (>100,000×). SEM uses a focused beam of high‐energy electrons to generate a variety of signals at the surface of solid samples. The incident electron beam is scanned in a raster pattern across the surface of the sample, and the electrons emitted are detected by an electron detector for each position in the scanned area.

### 3.3 Stability of particle size

**FIGURE 3:**
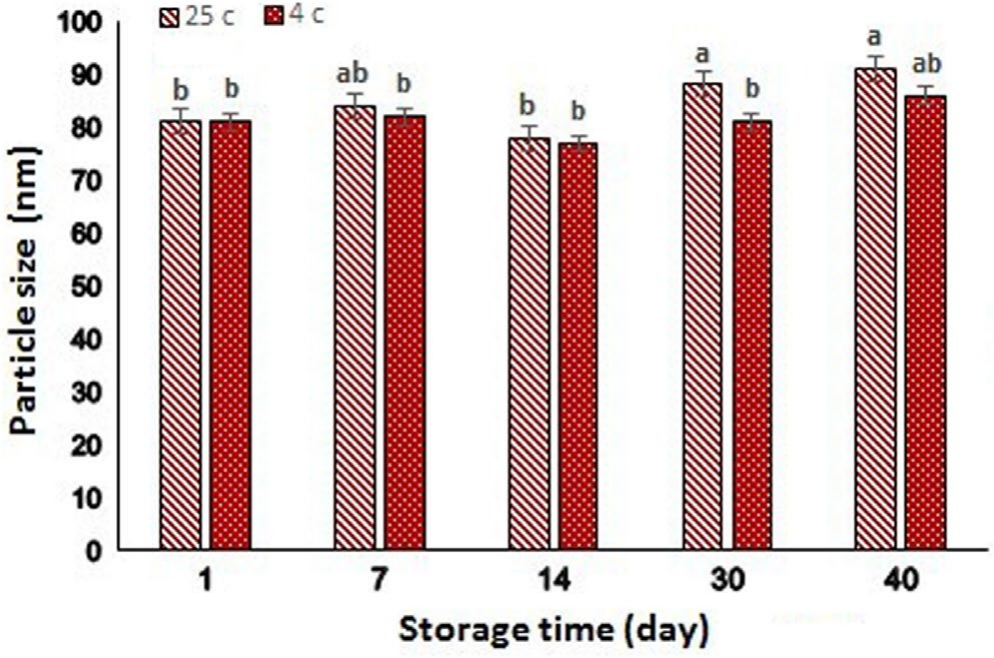
Stability of CLA loaded NLC formulations in storage time. (Different letters indicate significant differences at *p* < .05)

Over a 40‐day storage to period, the particle size of NLC stored at 22°C were similar to those stored at 4°C (Figure 3). NLC remained stable and uniform at both temperatures and the particle size of the system was not increased significantly (*p* > .05). The size distributions of both were monomodular. The presence of particles larger than 1 µm and a reduction in the particles number over time can show a physical instability.

Gravitational separations (Creaming or sedimentation), coalescence, and flocculation are main destabilization phenomena which affect the systems homogeneity (Araujo, Nikolic, Egea, Souto, & Garcia, 2011). A narrow particle size distribution minimizes the concentration gradient to the environment and inhibits the Ostwald ripening process (mass transfer from small particles to larger particles) (Talebi, Ghanbarzadeh, Hamishehkar, Pezeshki, & Ostadrahimi, 2019; Yang et al., 2018). According to obtained results, the CLA‐NLC can be stored at 4°C and 22°C, which are common conditions for the storage of foods and beverage.

### 3.4 Turbidity

**FIGURE 4:**
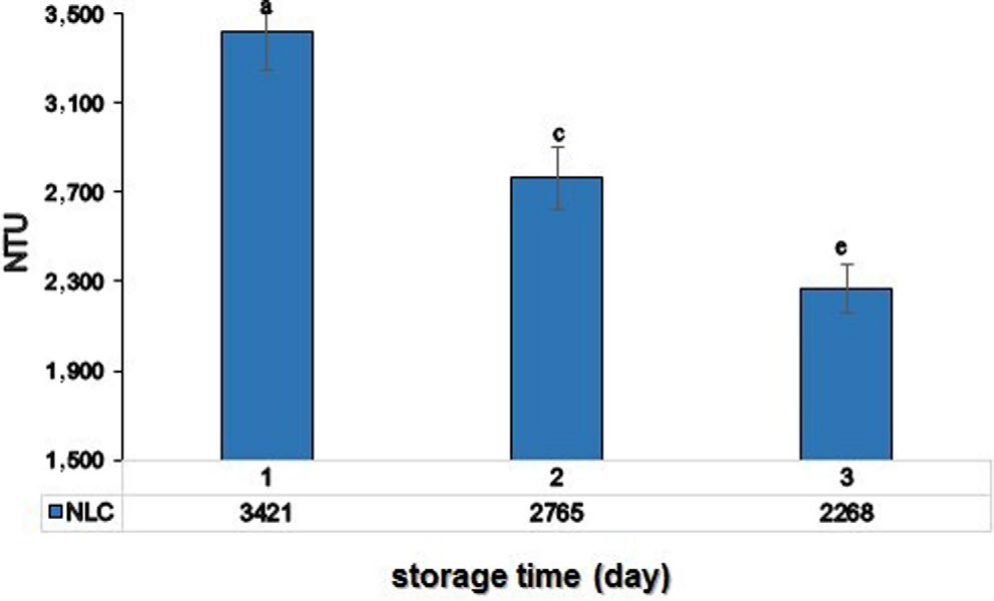
Turbidity of CLA loaded NLC formulations in 1st, 30th, and 60th day of storage. (Different letters indicate significant differences at *p* < .05)

There was no change of particle size after 60‐day period (Figure 4), so it can be said that systems turbidity decreased during storage time. The slight decrease in turbidity after 60 days of storage, could be due to the loss of components by the changes of mean particle size and gravitational separation of particles in solution and the flotation of particles to the suspension surface. Also, due to presence of solid lipid (CB) in NLC formulation, the turbidity of NLC formulations was higher (Figure 4), it is known that higher refractive index (RI) will produce systems with higher turbidity.

The physical destabilization of nanocarriers may be perceived by microscopy, spectroscopy, turbidity, and particle size analysis. Particle size reduction cause to an increase in solution clarity, colloidal stability, and also specific surface area which in turn increases the solubility and bioavailability (Keivani Nahr et al., 2018). The slight decrease in turbidity after 60 days of storage, could be due to the loss of components by the changes of mean particle size and gravitational separation of particles in solution and the flotation of particles to the suspension surface (Zhang, Bing, & Reineccius, 2016). The turbidity of NLC formulations was higher (Figure 4), it is known that higher refractive index (RI) will produce systems with higher turbidity. Since the RI of CB is 1.45 (Yunus, Fen, & Yee, 2009), these results would be expected. Considering that the usage of nanocarriers in beverages are naturally around 5% (Ni et al., 2015), the turbidity of CLA‐NLC in milk would be disregarded.

### 3.5 Oxidative stability of CLA

**FIGURE 5:**
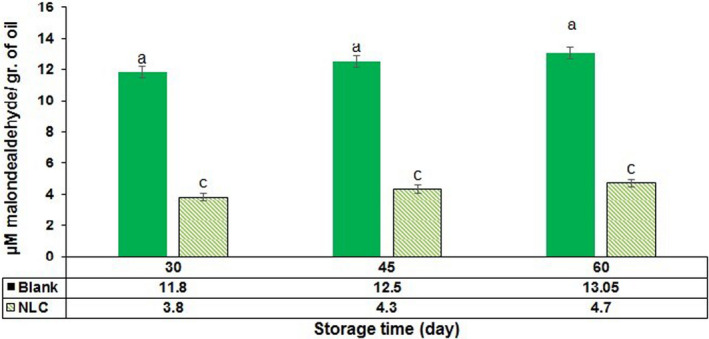
The result of thio barbioturic acid on witness samples (emulsion from 250 μg CLA in double‐distilled water) and NLC containing CLA in days 30, 45, and 60th of storage time. (Different letters indicate significant differences at *p* < .05)

According to Figure 5, less secondary oxidation products (malonealdehydes) created during storage time and a meaningful difference was observed among control sample and NLC in terms of the rate of generated malonealdehydes.

Oxidative stability increased by injection CLA in colloidal nanocarrier systems and protecting this compound against environmental conditions, light, and oxygen. So, less secondary oxidation products (malonealdehydes) created during storage time and a meaningful difference was observed among control sample and NLC in terms of the rate of generated malonealdehydes. Surfactant layer covers the small lipids and prohibits them from coalescence, thus the encapsulated material was preserved in the NLC (Pezeshki et al., 2014). It was in accordance with Nikbakht et al. (Nikbakht Nasrabadi, Goli, & Nasirpour, 2016) about emulsion preparation containing CLA. Malonealdehydes mainly create in auto‐oxidation with three or more double links. This compound is odorless and is able to contact with proteins.

### 3.6 FTIR analysis

**FIGURE 6:**
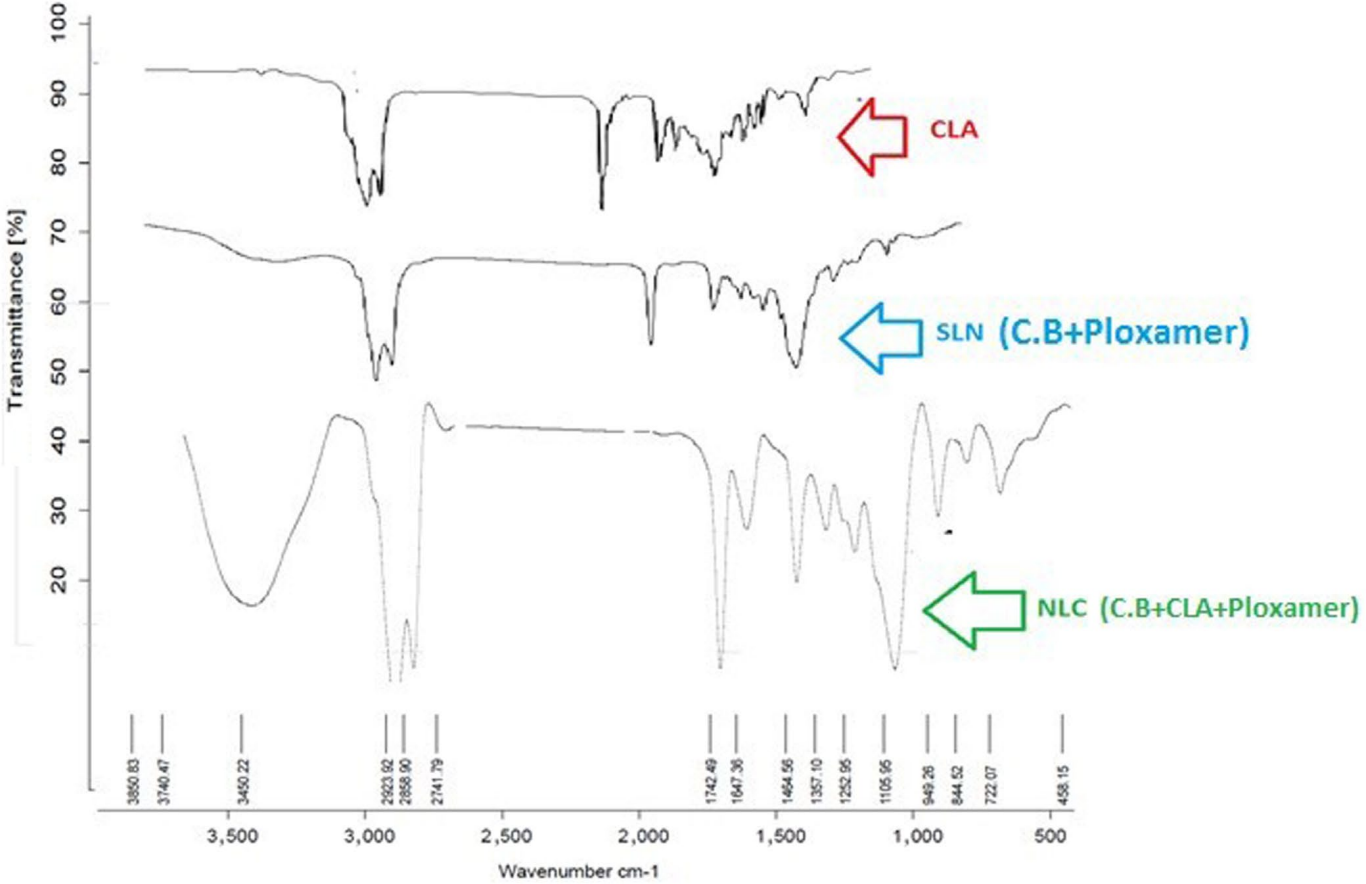
Fourier‐transform infrared (FTIR) spectra of NLC (CB + CLA + Ploxamer 407), SLN (CB + Ploxamer 407) and CLA

FTIR is a great technique of determining the interaction between components in the nanocarrier structure. FTIR can provide fundamental information on the molecular structure of organic and inorganic components (D'Angelo & Zodrow, 2011). FTIRIS offers unique possibilities to collect chemical information from biological samples with high spatial resolution (generally ~ 10 μm) (Lasch & Naumann, 2006). By comparing the peaks obtained from CLA, NLC and SLN, the increase in peak intensity in NLC containing CLA at 1,700 cm^−1^, related to the C꞊C‐H alkene group, weak vibrational peaks at 2,923 cm^−1^ of OH‐free CLA groups and the expanded hydroxyl groups in the NLC were shown in 3,450.22 cm^−1^, which is probably due to the binding with CLA in the NLC system (Figure 6). According to the present results, it can be shown the CLA in the NLC system without chemical bonding.

The results of FTIR analysis by other researchers also confirm the findings. Karimi et al. (2018) and Keivani Nahr et al. (2018) in the production of NLC containing turmeric extract and cardamom essential oil extract respectively, stated that the coated compound is compatible without the chemical reaction within the lipid matrix. Pezeshki et al. in their study on vitamin A—palmitate complex (2014) and beta carotene (2019) in the NLC system showed that these compounds are physically located within the NLC system and no chemical reaction has taken place between the components of the system and the active substance. Also, Sun et al. (2014), by examining the FTIR curve of the lipid nanocarriers containing resveratrol, by observing all the main peaks of resveratrol, showed that this substance was only molecularly distributed in the lipid matrix and no chemical reaction was observed between the lipid phase and resveratrol.

### 3.7 DPPH scavenging assay

The natural antioxidants have biological balance, so they are not accumulated in the body and cause the minimum side effects and tendency toward natural antioxidants has been increased (Hasrati, Govahi, & Mollaie, 2020). The antioxidant activity of NLC (at the concentration of 500 μg/ml) throughout the storage is presented in Table 2. By comparing the results of CLA and CLA encapsulated in NLC structure, there was a significant difference in the AA, which could be due to the CLA being free and exposed to environmental conditions (e.g. light and oxygen) and the degradation of the agent groups for the participate in AA and free radical scavenging. But, against the encapsulation of CLA in C.B and the protective role of surfactant layer around lipid particles in the NLC structure, the protection, and antioxidant properties were found to be higher. Furthermore, AA of NLC revealed no statistically significant change after 45 days of storage. Soleimanian et al. (2018) reported the similar results for AA by producing the NLC composed of solid lipids of bee wax, propolis wax, and pomegranate seed oil. There are numerous antioxidant methods for evaluation of antioxidant activity. For in vitro antioxidant screening, DPPH free radical scavenging, metal ion chelating, hydrogen peroxide scavenging, superoxide anion radical scavenging, and Ferric thiocyanate reducing activities are most commonly used. However, the total antioxidant activity of an antioxidant cannot be evaluated by using one single method, due to oxidative processes. Therefore, at least two methods should be employed in order to evaluate the total antioxidant activity (Gulsin, Alici, & Cesur, 2005). The model of scavenging the stable DPPH radical is a widely used method to evaluate antioxidant activities in a relatively short time compared with other methods. The effect of antioxidants on DPPH radical scavenging was thought to be due to their hydrogen donating ability. DPPH is a stable free radical and accepts an electron on hydrogen radical to become a stable diamagnetic molecule (Arulmozhi, Mazumder, Ashok, & Narayanan, 2007; Soares, Dinis, Cunha, & Almeida, 1997). Soleimanian et al. (2018) reported the similar results for AA by producing the NLC composed of solid lipids of bee wax, propolis wax, and pomegranate seed oil.

**TABLE 2:** Antioxidant activity of NLC and CLA during storage at different temperatures (4 and 22°C)

Formulation	Temperature	Antioxidant activity (%)			
		Day 0	Day 15	Day 30	Day 45
CLA‐NLC	22°C	32.72 ± 0.24Aa	31.33 ± 0.24ABa	30.32 ± 0.24ABa	29.42 ± 0.11Bb
	4°C	33.61 ± 0.13Aa	32.92 ± 0.84Aa	31.61 ± 0.14ABa	30.57 ± 0.18ABb
CLA	4°C	25.32 ± 0.22Cc	24.32 ± 0.22Cc	23 ± 0.22Cc	23.76 ± 0.22Cc
	22°C	23.32 ± 0.24Cc	22.32 ± 0.24Cc	21.98 ± 0.24Cc	21.12 ± 0.28Cc

Note

Different a, b, c letters in the same column and different A, B, C letters in the same row indicate a statistically significant difference (*p* < .05).

### 3.8 Protection of CLA in pasteurized low‐fat milk during storage

**FIGURE 7:**
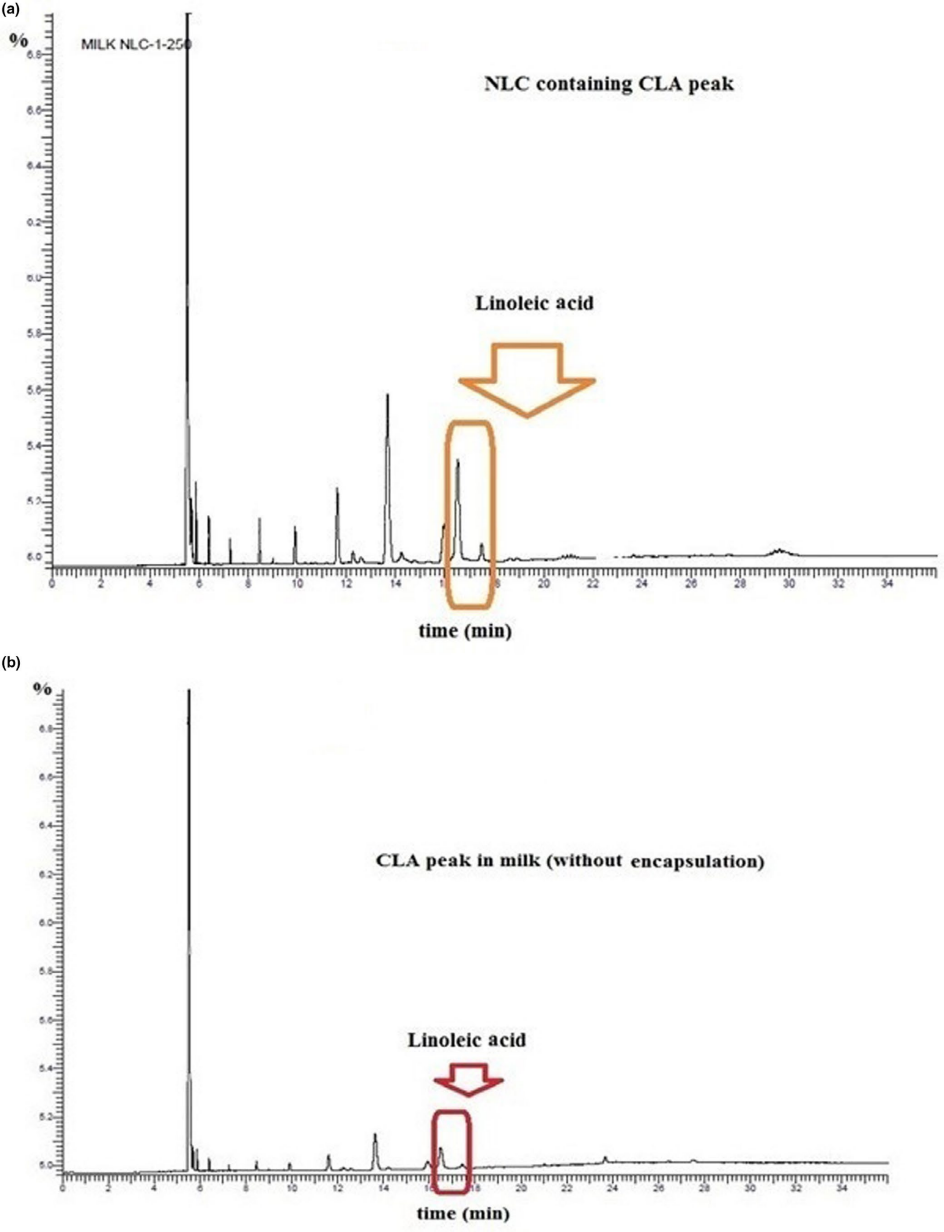
Chromatography results of fortified milk with (a) CLA‐NLC and (b) milk containing 250 μg pure CLA without encapsulation

CLA content in NLC‐enriched low‐fat milk was 3.9% of fatty acid in milk (regarding area peak (3.73) (Figure 7).

Considering NLC structure composed of solid lipid and liquid oil, a longer and more desirable release would be obtained from nanoparticles and it is possible to transmit active compound to aimed tissue. Also, the least distribution of encapsulated compound would be happened (Pezeshki et al., 2014; Shukla et al., 2014). Due to the presence of liquid oil in NLC formulation, compared to solid lipid nanoparticle structure (SLN) which only has solid lipid, outputting of active compound would lessen, highly EE %, protection of active compound, exposing it in environmental condition can be obtained (Karimi et al., 2018; Komaiko & McClements, 2014). Also, the presence of surfactant layer (Poloxamer 407) in NLC, intensively influenced the crystal behavior of lipid, and therefore, release of CLA to medium was less. The least distribution of encapsulated compound would be happened (Pezeshki et al., 2014; Shukla et al., 2014). Due to the presence of liquid oil in NLC formulation, compared to solid lipid nanoparticle structure (SLN) which only has soild lipid, outputting of active compound would lessen, highly EE %, protection of active compound, exposing it in environmental condition can be obtained (Karimi et al., 2018; Komaiko & McClements, 2014).

## 4 CONCLUSION

Food grade delivery systems may be used to contain lipophilic nutraceuticals in aqueous‐based foods and increase their bioavailability, functionality, and physical and chemical stability during the processing and storage time. NLC is a colloidal system that potentially can be used in fortification of food supplies. The particle size of NLC containing CLA was in nanoscale. They were stable during storage and unstable mechanism did not happen in them. By encapsulation CLA in NLC systems, the protection of CLA against oxidation was more and using NLC, reduced generating secondary oxidation products such as malonealdehydes. Also, stability of CLA in NLC formulation against thermal process, for example, pasteurization, environmental condition, and oxidation, was higher. Finally, using NLC can compensates the shortage of CLA in low‐fat milk which happens by removing and reducing fat content.

## CONFLICT OF INTEREST

The authors declare that they have no conflict of interest.

## ETHICAL APPROVAL

The authors declare no ethical issue related with this article.

## HUMAN AND ANIMAL STUDIES

This article is a scientific and research type, and has no human or animal examples.
